# Submicroscopic Burden of Zoonotic *Plasmodium knowlesi* Malaria on Mursala Island and *Plasmodium falciparum* and *Plasmodium vivax* Transmission in Mainland North Sumatra, Indonesia

**DOI:** 10.4269/ajtmh.25-0493

**Published:** 2025-12-04

**Authors:** Inke Nadia Diniyanti Lubis, Ranti Permatasari, Lambok Siahaan, R. Andika Dwi Cahyadi, Irbah Rhea Alvieda Nainggolan, Rycha Dwi Syafutri, Monica Nadya Sinambela, Silvia Jauharah, Agatha Lestari, Minerva Theodora, Hellen Prameswari, Kim A. Piera, Bridget E. Barber, Nicholas M. Anstey, Matthew J. Grigg

**Affiliations:** ^1^ Faculty of Medicine, Universitas Sumatera Utara, Medan, Indonesia;; ^2^ Duke–National University of Singapore, Singapore;; ^3^ Global and Tropical Health Division, Menzies School of Health Research and Charles Darwin University, Darwin, Australia;; ^4^ The Roslin Institute, Royal (Dick) School of Veterinary Studies, University of Edinburgh, Edinburgh, United Kingdom;; ^5^ National Malaria Control Programme, Directorate of Communicable Disease Control, Ministry of Health Republic of Indonesia, Jakarta, Indonesia;; ^6^ QIMR, Brisbane, Australia

## Abstract

Accurate molecular tools are essential for estimating zoonotic malaria transmission in Southeast Asia. This study applied ultrasensitive reverse-transcriptase real-time polymerase chain reaction (PCR) to detect zoonotic malaria in febrile patients from health facilities across three mainland districts (Batubara, Tanjung Balai, and Central Tapanuli) and separately on Mursala Island, North Sumatra, Indonesia. Among 64 participants on Mursala, 7 (10.9%) adults had *Plasmodium knowlesi* infections (including 5 agricultural workers and 2 adults with severe WHO anemia), and 2 (3.1%) adults had *Plasmodium vivax* infections. All were negative by microscopy and panparasite lactate dehydrogenase rapid diagnostic tests. No *P. knowlesi* infections were identified among 947 participants from mainland sites; PCR detected confirmed *Plasmodium* species in 26%, including *P. vivax* (17.5%) and *Plasmodium falciparum* (7.5%), with 30% of cases being submicroscopic. No *Plasmodium cynomolgi* infections were identified. *Plasmodium knowlesi* transmission is low in North Sumatra; however, it may cause WHO-defined severe malaria. Molecular diagnostics remain crucial for identifying zoonotic malaria and should be integrated into surveillance systems to inform public health control measures.

## INTRODUCTION

Zoonotic malaria caused by *Plasmodium knowlesi* has emerged as a significant public health challenge in Southeast Asia.^1^^,^^2^ Indonesia has a sizeable population at risk of zoonotic malaria^3^^,^^4^ because of its ecological diversity and widespread overlap between the parasite’s major natural macaque hosts (*Macaca fascicularis* and *Macaca nemestrina*) and *Anopheles leucosphyrus* group vectors.^5^ Rapid agricultural expansion and land use changes have amplified the risk of zoonotic malaria by increasing interaction between humans, macaques, and mosquito vectors.^6^^,^^7^ The risk is particularly high among adults engaged in forest and agricultural activities.^8^^,^^9^ However, understanding of *P. knowlesi* transmission in Indonesia is limited because of being frequently misdiagnosed as nonzoonotic species using routine microscopy, with *Plasmodium falciparum* and *Plasmodium vivax* often incorrectly identified even more so than the morphologically similar *Plasmodium malariae*.^10^^,^^11^ Lateral flow-based rapid diagnostic tests (RDTs), designed and commonly used for detection of *P. falciparum* and *P. vivax*, have also lacked the specificity and until recently, the sensitivity required for reliable *P. knowlesi* identification, particularly at low parasite densities.^12^^–^^14^ Diagnostic misclassification using available point-of-care diagnostics has previously obscured the actual burden of *P. knowlesi* malaria, a trend first observed in Sarawak, Malaysian Borneo, where a rise in cases of microscopy-diagnosed *P. malariae* ultimately led to the initial molecular identification of *P. knowlesi* as a dominant cause of human malaria in 2004.^15^

The use of accurate polymerase chain reaction (PCR) assays in research settings has highlighted the presence of *P. knowlesi* transmission across western Indonesia, particularly within the provinces of Sumatra^10^^,^^11^^,^^16^^–^^21^ and Kalimantan,^22^^–^^26^ but outside the nonendemic eastern province of Papua, where the highest burden of nonzoonotic malaria remains.^27^ However, there remain significant gaps in our understanding of the distribution of *P. knowlesi* within areas such as North Sumatra, where diverse ecological conditions are conducive to zoonotic malaria transmission^3^ but where many districts have not been systematically evaluated using sensitive molecular tools. *Plasmodium knowlesi* transmission has been reported to date in the districts of Batubara, Langkat, and Dairi and the islands of South Nias.^11^^,^^20^^,^^28^ Other zoonotic malaria species, such as *Plasmodium cynomolgi*, have not been screened for in western Indonesia, despite sharing the same macaque hosts and mosquito vectors.^29^ Understanding the prevalence and distribution of zoonotic *Plasmodium* species in regions like North Sumatra is critical for improving malaria control efforts, meeting WHO malaria elimination goals, and addressing the hidden burden of zoonotic malaria in Indonesia.^30^ The aim of this study was to conduct molecular epidemiological surveillance using improved ultrasensitive PCR for both zoonotic *P. knowlesi* and *P. cynomolgi* detection in high-risk areas across North Sumatra.

## MATERIALS AND METHODS

### Study design.

The main study design consisted of longitudinal passive malaria case detection of febrile patient presentations at selected health facilities on mainland North Sumatra, Indonesia. A separate small cross-sectional study was conducted on Mursala Island.

### Study sites.

**Figure 1.**
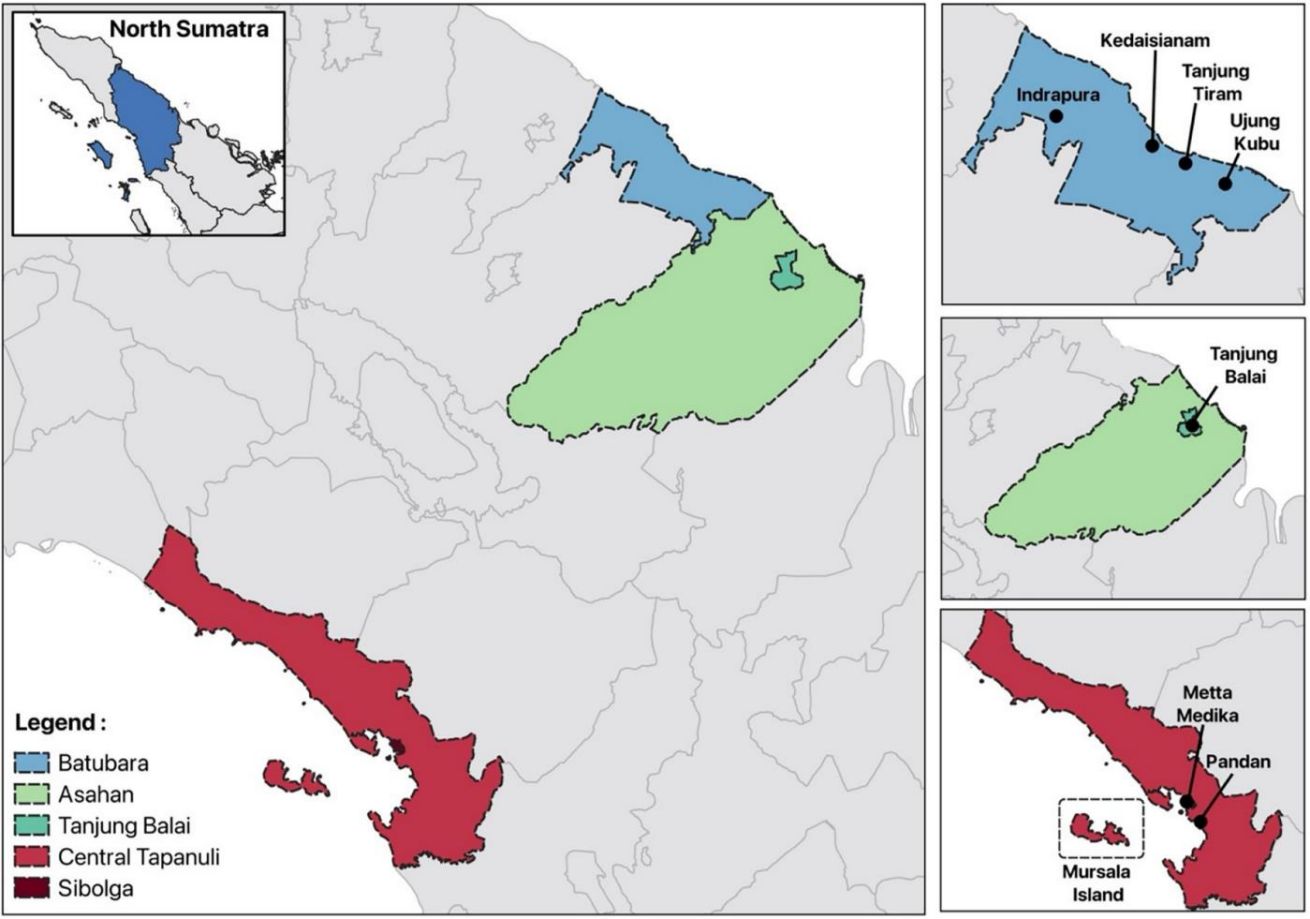
Map of study sites: Batubara district (blue), Tanjung Balai district (green), and Central Tapanuli (red).

The health facility surveys on the North Sumatra mainland covered a study area of 6,933 kilometers squared (km^2^), with an estimated 1,579,000 people at risk^31^ (Figure 1). There were eight health facilities from across three districts, including four government primary health clinics in Batubara, two primary health clinics and a referral hospital in Central Tapanuli, and an urban referral hospital in Tanjung Balai. The estimated prevalence of malaria was taken into account when selecting the health facility locations. At the time of the study design in 2019, provincial reports indicated that Batubara accounted for 41% of malaria cases in North Sumatra, with an annual parasite index (API) of 1.1 positive case per 1,000 people tested; Asahan accounted for 10% prevalence, with an API of 0.16, and Central Tapanuli accounted for 2% prevalence, with an API of 0.06.^1^^,^^3^

A separate cross-sectional study was performed on Mursala Island, which is also part of Central Tapanuli district; it is located 22 km off the coast and inhabited by 5,400 people, and it has a size of 80 km^2^. Community approval and awareness for the survey were obtained after meeting with village leaders and heads of households.

### Study participants.

Patients at mainland study health facilities were enrolled if they met the following inclusion criteria: age of 1 year old or older, body temperature above 37.5°C or a history of fever in the last 48 hours, presented with nonspecific symptoms (suspected malaria), resided primarily in the study district within the previous 1 year, and provided informed written consent (from parents/guardians if younger than 18 years old). For Mursala Island, criteria were similar, although a history of fever was not required. Demographic, clinical, and environmental data and malaria prevention methods and activities, including macaque interaction data, were collected by trained research staff using a standardized electronic case record form (REDCap v. 10.6, Nashville, TN). Severe malaria was defined using WHO 2014 Research and Epidemiological criteria for severe disease.^32^

### Blood sampling.

Venous whole blood (or a capillary sample in those 10 years old or younger and all participants from Mursala) was collected in ethylenediaminetetraacetic acid for standard hematology and biochemistry at local laboratories where available. Hemoglobin was measured with a HemoCue^®^ 201 machine (HemoCue AB, Ängelholm, Sweden) on Mursala Island. Malaria screening was performed using Ministry of Health-supplied routine malaria RDTs with combined pan-*Plasmodium* species parasite lactate dehydrogenase (PAN-pLDH) and *P. falciparum*-specific histidine-rich protein-2 (Pf-HRP2) targets depending on site availability and microscopic examination of blood smears stained with Giemsa. Slides and RDTs were read by local health facility officers, and slides were re-examined and quantified by an experienced microscopist blinded to the first microscopy and RDT results according to standard WHO procedures.^33^ A total of 300 and 100 *µ*L of whole blood from adults and children, respectively, were placed in field-stable DNA/RNA Shield^TM^ (Zymo Research, Irvine, CA) total nucleic acid preservation media and frozen at 4°C before shipping to the Faculty of Medicine, Universitas Sumatera. Patients confirmed as malaria positive on point-of-care testing were treated according to national guidelines.^34^

### Laboratory malaria PCR detection.

Total nucleic acids were extracted (QIAamp DNA Blood Mini Kit, Hilden, Germany) from 200 *µ*L of the blood sample preserved in DNA/RNA Shield followed by high-capacity complementary DNA (cDNA) reverse transcription (Applied Biosystems, Waltham, MA). Amplification of cDNA was conducted using a quantitative real-time polymerase chain reaction (qRT-PCR) workflow targeting 18S ribosomal RNA genes.^35^ Positive *Plasmodium* genus results were defined as cycle threshold (Ct) values below 40 from duplicate runs, with a Ct value difference of less than three. Those positive had conventional species-specific PCR assays performed using the same cDNA for *P. knowlesi*,^36^ *P. cynomolgi*,^37^ and other human *Plasmodium* species (*P. falciparum*, *P. vivax*, *Plasmodium ovale* spp., and *P. malariae*^38^) detection. Each PCR amplification included *Plasmodium* species positive and negative controls and molecular weight standards.

## STATISTICAL ANALYSES

All statistical analyses were performed using Stata v. 17.0 (StataCorp, College Station, TX). The χ^2^ or Fisher exact tests were used to evaluate proportional differences in binary variables, and the Student’s *t*-tests or Wilcoxon rank sum tests were used for pair-wise comparisons of clinical and epidemiological data. Results of microscopy and RDT assays were evaluated against reference PCR, enabling calculation of diagnostic sensitivity and specificity with exact binomial 95% CIs. The crude malaria incidence rate was calculated as the number of malaria cases per 100,000 at-risk people per year at a district catchment level. Study site district catchment populations were projected estimates from 2020 census data.^39^ Logistic regression models were used for univariable and multivariable epidemiological associations with individual *Plasmodium* species infections, with odds ratios (ORs) and 95% CIs reported.

## RESULTS

### Mainland health facility surveys.

**Figure 2.**
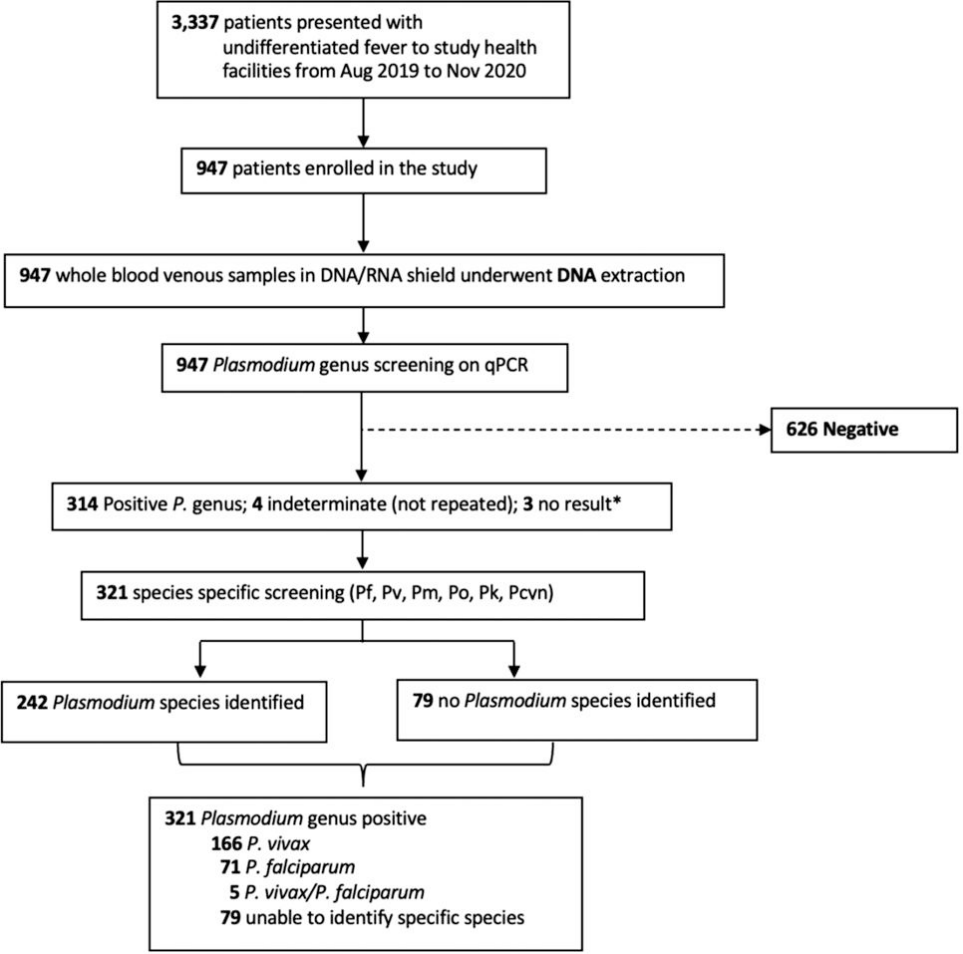
Enrollment flowchart for health facility surveys and *Plasmodium* (*P*.) species results. Aug = August; Nov = November; Pcyn = *Plasmodium cynomolgi*; Pf = *Plasmodium falciparum*; Pk = *Plasmodium knowlesi*; Pm = *Plasmodium malariae*; Po = *Plasmodium ovale*; Pv = *Plasmodium vivax*; qPCR = quantitative polymerase chain reaction.

From August 2019 to December 2020, there were 3,377 patients with acute febrile illness presenting to the health facilities in mainland North Sumatra (Figure 2). Of these, 947 (28.1%) were enrolled, including 704 (74.3%) patients from Batubara, 136 (14.4%) patients from Tanjung Balai, and 107 (11.3%) patients from Central Tapanuli. The median age was 17 years old (interquartile range [IQR]: 12–34; range: 2–67), and 48% were female.

Overall, 321 (33.9%; 95% CI: 30.9–37.0%) patients tested positive for malaria by PCR; 71 (7.5%) patients tested positive for *P. falciparum*, 166 (17.5%) patients tested positive for *P. vivax*, and 5 (0.5%) patients tested positive for mixed *P. falciparum*/*P. vivax*. In 79 (8.3%) patients, we were unable to determine the exact *Plasmodium* species infection. There were 96 febrile patients with submicroscopic infections (29.9% of total infections; 95% CI: 24.9–35.2%), including 8 patients with *P. falciparum*, 16 patients with *P. vivax*, and 72 patients with *Plasmodium* genus.

There were no significant differences in the median age (approximately 16 years old) or the proportion of children (approximately 35%) with *P. falciparum* or *P. vivax* infections (Table 1). The majority presented with nonspecific symptoms, most commonly headache, nausea/vomiting, cough, and abdominal pain, with a median preceding fever duration of 6 days. Anemia (using WHO age and sex hemoglobin criteria^40^) was observed in more than 40% of patients infected with *P. falciparum*, *P. vivax*, or undetermined *Plasmodium* species. Thrombocytopenia (platelet count <150 × 10^3^/*µ*L) was present in more than 75% of patients with *P. falciparum* or *P. vivax* malaria. In contrast, anemia and thrombocytopenia were only present in 30% and 22% of the febrile nonmalaria controls, respectively (*P* ≤0.008). A previous history of malaria was positively associated with acute infections (OR: 2.91; 95% CI: 1.88–4.50; *P* <0.001).

**Table 1:** Clinical and laboratory features of *Plasmodium* species infections in mainland North Sumatra

Patient Characteristics	North Sumatra—Mainland Sites					
	*Plasmodium falciparum*	*Plasmodium vivax*	*Plasmodium falciparum/ Plasmodium vivax*	*Plasmodium* genus	Control (negative)	*P*-Value (across groups)
No. enrolled	71	166	5	79	626	
Age, median years	16	16	18	15	18	0.063
IQR	12	13	14	17	26	
Range	2–55	4–65	4–30	2–54	1–80	
Children (age younger than 15 years old), *n* (%)	25 (35.2)	58 (34.9)	2 (40)	37 (46.8)	229 (36.6)	0.46
Male gender, *n* (%)	38 (53.5)	100 (60.2)	4 (80)	33 (41.8)	321 (51.3)	**0.035**
Previous malaria (self-reported), *n* (%)	15 (21.1)	31 (18.7)	2 (40)	5 (6.3)	40 (6.4)	**<0.001**
Symptoms on enrolment, *n* (%)						
Headache	70 (98.6)	147 (88.6)	4 (80)	62 (78.5)	503 (80.4)	**<0.001**
Nausea	54 (76.1)	127 (76.5)	4 (80)	51 (64.6)	422 (67.4)	0.069
Cough	41 (57.7)	75 (45.2)	2 (40)	40 (50.6)	324 (51.8)	0.45
Shortness of breath	11 (15.5)	17 (10.2)	0 (0)	15 (18.9)	140 (22.4)	**0.005**
Vomiting	37 (52.1)	75 (45.2)	2 (40)	29 (36.7)	276 (44.1)	0.432
Abdominal pain	36 (50.7)	60 (36.1)	4 (80)	39 (49.4)	313 (50)	**0.014**
Diarrhea	8 (11.3)	17 (10.2)	0 (0)	6 (7.6)	68 (10.9)	0.921
Bleeding	0 (0)	5 (3.0)	0 (0)	1 (1.3)	4 (0.6)	0.106
Convulsions	10 (14.1)	13 (7.8)	0 (0)	4 (5.1)	118 (18.8)	**<0.001**
Fever (temperature >37.5°C), *n* (%)	35 (49.3)	101 (60.8)	2 (40)	51 (64.6)	293 (46.8)	**0.001**
Febrile days (before presentation), median (IQR, range)	6 (4, 1–40)	6 (4, 1–30)	7 (7, 5–15)	5 (3, 2–25)	5 (4, 0–40)	
Microscopy result*						
* Plasmodium vivax*	–	99	2	2	11	–
* Plasmodium falciparum*	42	2	1	2	10	–
* Plasmodium falciparum/Plasmodium vivax*	20	44	2	3	7	–
* Plasmodium falciparum/Plasmodium knowlesi*	1	–	–	–	–	–
* Plasmodium knowlesi/Plasmodium malariae*	–	1	–	–	–	–
Negative	8	20	–	72	598	–
Sensitivity, % (95% CI)	59.2 (46.8–70.7)	59.6 (51.8–67.2)	60 (14.7–94.7)	–	95.5 (93.6–97)	
Specificity, % (95% CI)	98.4 (97.3–99.1)	98.1 (96.9–98.9)	82.2 (79.6–84.6)		68.8 (63.5–73.9)	
Parasite count						
Positive result, *n* (%)	62 (87)	145 (87)	5 (100)	7 (8.9)	–	
Parasites/*µ*L, geom. mean (95% CI)	3,309 (2,060–5,315)	4,199 (3,510–5,023)	8,010 (4,139–15,502)	7,026 (3,330–14,822)	–	
Parasites/*µ*L, median	4,870	5,009	9,638	4,525	–	
IQR	1,330–11,673	2,019–9,017	7,068–13,007	3,668–14,136	–	
Range	8–53,850	200–54,800	2,340–15,904	2,305–36,280	–	
RDT result^†^ (PAN-pLDH/Pf-HRP2)						
No. conducted, *n* (%)	51 (72)	123 (74)	3 (60)	72 (91)	441 (71)	
Sensitivity, % (95% CI)	82.4 (69.1–91.6)	81.3 (73.3–87.8)	100 (29.2–100)	11.1 (4.9–20.7)	–	
Specificity, % (95% CI)	78.5 (75.1–81.6)	86.0 (82.9–88.8)	73.9 (70.5–77.2)	71.8 (68.1–75.4)	93.4 (90.7–95.6)	
Anemia^‡^ (baseline), *n*/*N* (%)	20/43 (47)	41/92 (45)	3/4 (75)	15/37 (41)	120/398 (30)	**0.008**
Creatinine^§^ (*µ*mol/L), median (IQR)	106 (92–124)	115 (97–133)	115 (102–155)	97 (94–115)	97 (87–115)	0.003
Platelet count, ×10^3^/*µ*L, median (IQR) [95% CI]	102 (55–137)[12–425]	98 (68–146)[11–565]	75 (58–133)[50–266]	227 (148–309)[11–520]	237 (162–315)[10–758]	**<0.001**
Thrombocytopenia (platelet count <150 × 10^3^/*µ*L), *n*/*N* (%)	36/42 (86)	69/92 (75)	3/4 (75)	10/37 (27)	87/399 (22)	**<0.001**
Severe malaria,^¶^ *n* (%) [95% CI]	0 (0)	1 (0.6) [0–3.3]	0 (0)	0 (0)	–	0.244

geom. = geometric; IQR = interquartile range; PAN-pLDH = pan-*Plasmodium* species parasite lactate dehydrogenase; Pf-HRP2 = *Plasmodium falciparum*-specific histidine-rich protein-2; RDT = rapid diagnostic test. Control indicates that the participant was malaria negative on polymerase chain reaction with acute febrile illness. Bold formatting was used to indicate statistically significant *P*-values.

* Sensitivity/specificity calculated for *Plasmodium* species monoinfection versus non-*Plasmodium* species detection on microscopy against reference polymerase chain reaction.

^†^ Sensitivity/specificity calculated for PAN-pLDH detection for *Plasmodium knowlesi* versus non-*Plasmodium knowlesi* on reference polymerase chain reaction.

^‡^ Anemia based on WHO 2011 hemoglobin measurement criteria.

^§^ Creatinine measurement obtained in 36% of malaria cases and 49% of controls.

^¶^ Severe malaria defined using WHO 2014 Research and Epidemiological criteria.

There was a single patient meeting 2014 research and epidemiological criteria for WHO-defined severe disease (0.4% of malaria cases with an identified *Plasmodium* species; 95% CI: 0.01–2.3%): an adult male with vivax malaria, a hemoglobin of 6.3 g/dL, and a parasite count of 3,120/*µ*L.

On routine microscopy, 249 positive results for malaria were reported, including 12% with *P. vivax*, 6% with *P. falciparum*, and a single *P. knowlesi*/*P. malariae* result. These findings resulted in an overall sensitivity for *Plasmodium* species detection against reference PCR of 68.8% (95% CI: 63.5–73.9%), with a specificity of 95.5% (95% CI: 93.6–97%) against malaria-negative controls. *Plasmodium falciparum* single infections were correctly identified in 42 (59.2%) of patients, with a further 21 cases (29.6%) misidentified as mixed infections. Similarly, single *P. vivax* infections were correctly identified in 60% of participants; however, they were misidentified as mixed *P. vivax* infections in another 27%. In contrast, there were only small numbers of false-positive microscopy results for *P. falciparum* and *P. vivax* single infections, leading to high diagnostic specificities of 98.4% (95% CI: 97.3–99.1%) and 98.1% (95% CI: 96.9–98.9%) for each species, respectively. The geometric mean parasite count for *P. falciparum* infections was low at 2,844/*µ*L (95% CI: 2,055–3,936/*µ*L), similar to *P. vivax* at 2,273/*µ*L (95% CI: 2,487–3,187/*µ*L).

The performance of the combined PAN-pLDH/Pf-HRP2 RDT was poor compared with reference PCR, with 155 (22.5%) positive PAN-pLDH results and 79 (11.4%) positive Pf-HRP2 results recorded. These findings resulted in sensitivity and specificity of 82.4% (95% CI: 69.1–91.6%) and 78.5% (95% CI: 75.1–81.6%), respectively, for *P. falciparum* detection and sensitivity and specificity of 81.3% (95% CI: 73.3–87.8%) and 86.0% (95% CI: 82.9–88.8%), respectively, for *P. vivax*. Sensitivity of the RDT compared with microscopy against PCR as the reference was higher overall for detection of *P. falciparum* (82% versus 53%; *P* = 0.0015) and *P. vivax* (81% versus 65%; *P* = 0.0065) single infections.

**Figure 3.**
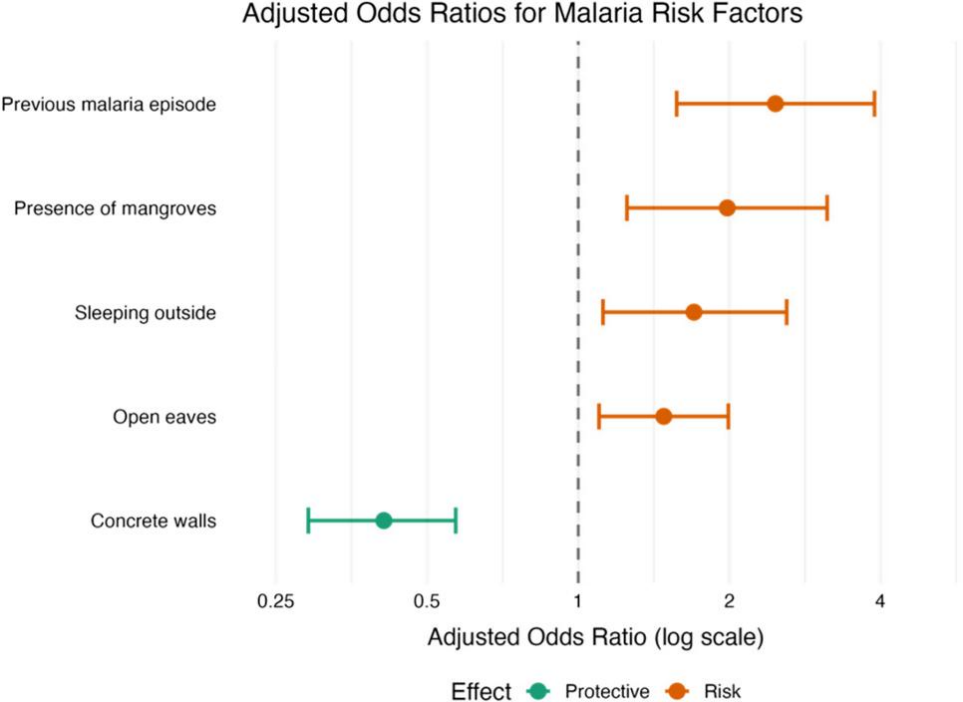
Forest plot of adjusted odds ratios for epidemiological associations with malaria acquisition risk.

Student was the most frequently reported primary occupation among malaria cases, accounting for approximately 50% overall (Supplemental Table 1), which reflects the predominantly adolescent cohort. On univariable analysis, a history of sleeping outdoors was associated with higher malaria risk, whereas insecticide-treated bed net use and personal insect repellent were protective. Housing type also played a key role; concrete walls were protective, whereas open eaves increased risk. Proximity to mangroves or cleared forest areas was also associated with higher odds of infection. Factors that remained independently associated with malaria in the multivariable analysis are summarized in Figure 3.

### Mursala Island cross-sectional survey.

In a small cross-sectional survey conducted on Mursala Island in September 2019, 64 individuals were enrolled (Table 2). The median age was 27 years old (IQR: 15.5–40; range: 1–80), and 58% were female. Most participants (92%) were symptomatic, including 35% reporting a history of fever. Polymerase chain reaction detected *Plasmodium* spp. infections in nine individuals (14.1%; 95% CI: 6.6–25%), comprising six with *P. knowlesi*, one with *P. vivax*, one with mixed *P. knowlesi/P. vivax*, and one *Plasmodium* genus-positive case with undetermined species. No infections were detected in patients younger than 18 years of age. None of the *Plasmodium* species-infected individuals reported a prior malaria diagnosis.

**Table 2:** Clinical and laboratory features of *Plasmodium* species infections in Mursala Island

Patient Characteristic	North Sumatra—Mursala Island				
	*Plasmodium knowlesi*	*Plasmodium vivax*	*Plasmodium knowlesi/ Plasmodium vivax*	Control	*P*-Value (*Plasmodium knowlesi* vs. control)
No. enrolled, *n* (%)	6 (10)	1 (2)	1 (2)	55 (86)	–
Age, years					
Median	46	34	26	25	**0.025**
IQR	30–60	–	–	14–40	
Range	23–72	–	–	1–80	
Children (age younger than 15 years old), *n* (%)	0 (0)	0 (0)	0 (0)	14 (25)	0.321
Male gender, *n* (%)	3 (50)	0 (0)	0 (0)	24 (44)	0.999
Previous malaria (self-reported), *n* (%)	0	0	0	16 (29)	0.326
Symptoms on enrolment, *n* (%)					
Any symptom	6 (100)	1 (100)	1 (100)	50 (91)	0.999
Fever history (within 48 hours)	2 (30)	1 (100)	0	31 (9)	
Headache	6 (100)	1 (100)	0	42 (12)	
Nausea	3 (50)	0	1 (100)	21 (6)	
Cough	6 (100)	0	0	35 (10)	
Shortness of breath	4 (75)	1 (100)	1 (100)	17 (5)	
Vomiting	0 (0)	0	0	15 (4)	
Abdominal pain	5 (83)	1 (100)	0	31 (9)	
Fever (temperature >37.5°C)	0	0	0	0	–
Febrile days (before presentation), *n* (IQR) [range]	4.5 (2–7) [2–7]	14	–	7 (2–7) [1–30]	0.670
Microscopy result—positive	0 (0)	0 (0)	0 (0)	0 (0)	–
RDT result (PAN-pLDH/Pf-HRP2)	0 (0)	0 (0)	0 (0)	0 (0)	–
Anemia* (baseline), *n*/*N* (%)	3/3 (100)*	0/1 (0)	1/1 (100)	15/41 (37)	0.062

IQR = interquartile range; PAN-pLDH = pan-*Plasmodium* species parasite lactate dehydrogenase; Pf-HRP2 = *Plasmodium falciparum*-specific histidine-rich protein-2; RDT = rapid diagnostic test.

* Two adult patients—one with *Plasmodium knowlesi* infection and another with mixed *P. knowlesi/P. vivax* infection—had hemoglobin levels of 5.1 g/dL and 4.4 g/dL, respectively, on HemoCue testing, according to WHO age- and sex-based criteria^38^).

Overall, seven patients (10.9%; 95% CI: 4.7–21.9%) were infected with *P. knowlesi*, with a higher median age (36 years old; IQR: 26–60) compared with malaria-negative or non-*P. knowlesi* cases (17 years old; *P* = 0.037). Females accounted for 57% of *P. knowlesi* cases. Common symptoms among *P. knowlesi* cases included headache, cough, and abdominal pain, although only 29% reported fever in the preceding 48 hours. The median reported duration of fever was 4.5 days (IQR: 2–7) for *P. knowlesi* and 14 days for *P. vivax*. Anemia, defined by WHO age- and sex-specific thresholds,^40^ was present in all *P. knowlesi*-infected individuals tested. Two adults with *P. knowlesi* infections met WHO criteria for severe malaria,^32^ with hemoglobin levels of 4.4 and 5.1 g/dL, respectively. Compared with nonmalaria controls, *P. knowlesi* cases had significantly lower median hemoglobin levels (7.1 versus 13 g/dL; *P* = 0.004). No other severe malaria features were observed. However, further assessment of respiratory distress or organ dysfunction (e.g., acute kidney injury, hyperbilirubinemia, hypoglycemia, and metabolic acidosis) was limited by the lack of appropriate clinical and laboratory facilities, respectively. None of the *P. knowlesi* or *P. vivax* infections were detected by microscopy or the combined PAN-pLDH/Pf-HRP2 RDT.

In Mursala, although the small survey size limits meaningful comparisons, five of the seven individuals infected with *P. knowlesi* had occupations related to agriculture (four farmers and one plantation worker) (Supplemental Table 2). Recent forest exposure (>4 hours) and proximity to rubber plantations were more common among *P. knowlesi* cases (86% each) than controls (46% and 43%, respectively; *P* = 0.076 for both comparisons). However, forest activities related to wood collection were associated with higher risk of *P. knowlesi* acquisition (OR: 16; 95% CI: 1.8–144; *P* = 0.013). Bed net use the night before enrollment was common in both groups (86% of *P. knowlesi* cases versus 64% of controls). Only one *P. knowlesi*-infected case reported sleeping outside in the previous 2 weeks. Regular awareness of monkey presence was high among cases (86%), although not significantly different from controls. No household structural or environmental factors, including wall type, elevation, or open eaves, were significantly associated with infection.

## DISCUSSION

This study aimed to further characterize the distribution of zoonotic *P. knowlesi* infections in North Sumatra, Indonesia using accurate molecular tools. Key findings include the presence of previously unreported *P. knowlesi* submicroscopic symptomatic infections on the remote rural island of Mursala. Additionally, results highlight the significant ongoing burden of nonzoonotic malaria (34%) detected by PCR among acute febrile illness presentations in densely populated mainland coastal areas. North Sumatra public health reporting estimated that *P. falciparum* caused 15% of clinical malaria and that *P. vivax* caused 85% of clinical malaria in 2019 (Provincial Health Office 2019, unpublished data). In our study, the predominance of *P. vivax* infections underscores the ongoing contribution of this species to the malaria burden despite national elimination efforts, with a doubling of malaria cases reported in North Sumatra since the coronavirus disease 2019 (COVID-19) pandemic in 2020.^27^ Although Mursala Island had a lower overall malaria prevalence, submicroscopic infections were predominantly caused by zoonotic *P. knowlesi* transmission. No other zoonotic *Plasmodium* species, such as *P. cynomolgi*, were identified.

Although *P. knowlesi* infections have been previously reported in North Sumatra,^11^^,^^20^ none were detected during this study among participants from densely populated coastal mainland sites located away from higher-risk agricultural or forest areas. Modeled transmission suitability for *P. knowlesi* in the coastal districts of Batubara and Central Tapanuli is relatively low, with median values of 0.12 (IQR: 0.05–0.38) and 0.35 (IQR: 0.13–0.55), respectively, compared with 0.61 (IQR: 0.27–0.82) in the more interior region of South Tapanuli (R. J. Tobin, L. E. Harrison, M. K. Tully, I. N. D. Lubis, R. Noviyanti, N. M. Anstey, G. S. Rajahram, M. J. Grigg, J. A. Flegg, D. J. Price, and F. M. Shearer, unpublished data).^3^ This finding likely reflects distinct epidemiological patterns, similar to those in Malaysian Borneo, where heterogeneous *P. knowlesi* transmission is concentrated in rural areas undergoing land use change.^3^^,^^41^^,^^42^ Unlike Malaysia, however, there is limited understanding about the distribution and behavior of suspected *An. leucosphyrus* group mosquito vectors across Sumatra^43^^,^^44^ or the variation in *P. knowlesi* infection prevalence in local macaque populations,^21^^,^^44^^,^^45^ both of which are key determinants of spatial zoonotic transmission patterns.^3^^,^^5^^,^^46^ However, the demographic profile of *P. knowlesi*-infected individuals—exclusively adults—suggests higher exposure among those engaged in agricultural activities where macaque hosts are present. Both males and females had *P. knowlesi* infections detected, with similar self-reported exposure patterns aligning with findings from other areas of previously reported zoonotic malaria transmission in Malaysia^9^ and elsewhere in western Indonesia.^16^ In contrast, *P. falciparum* and *P. vivax* infections in this study predominantly affected younger adolescents, consistent with well-established demographic patterns in other endemic regions approaching elimination of these nonzoonotic species.^47^

The majority of zoonotic and nonzoonotic malaria cases presented with nonspecific clinical symptoms, highlighting the ongoing disease burden of malaria and critical need for vigilant point-of-care diagnostic testing. Laboratory findings of moderate anemia and thrombocytopenia were more common in malaria-positive patients, which may aid clinical suspicion for diagnostic screening. The small number of patients with *P. knowlesi* infections from Mursala all exhibited anemia, despite extremely low-level submicroscopic parasitemia, including two cases that met WHO criteria for severe malarial anemia (hemoglobin <7g/dL).^32^ Although chronic causes of anemia cannot be excluded—particularly given the high baseline prevalence in malaria-negative individuals—*P. knowlesi*-associated anemia may also result from splenic retention of less-deformable uninfected erythrocytes and/or bystander destruction of uninfected erythrocytes or dyserythropoiesis,^48^^,^^49^ potentially exacerbating underlying vulnerability. Evidence from Malaysia similarly reports anemia in uncomplicated *P. knowlesi* infections, although generally less prevalent and severe than in *P. falciparum* and *P. vivax* malaria.^50^^,^^51^ The low parasitemia levels in Mursala cases could reflect partial immunity from repeated *P. knowlesi* exposure, crossprotection from prior *P. vivax* infections, or reduced erythrocyte invasion because of parasite or host genetic factors, including hemoglobinopathies.^52^ Parasite counts of >15,000/*µ*L have been linked to a >16-fold risk of severe *P. knowlesi* malaria in Malaysian Borneo, often with clinical complications, such as jaundice, respiratory distress, hypotension, and acute kidney injury.^50^^,^^51^ These manifestations were not observed either because of the low parasite counts or because of the limited laboratory testing available in this study.

The threat of emerging *P. knowlesi* transmission highlights the ongoing importance of access to robust diagnostic capabilities and antimalarial treatment in provinces approaching elimination of nonzoonotic *Plasmodium* species. Indonesia’s national malaria control program currently relies on passive surveillance using microscopy or PAN-pLDH/Pf-HRP2-based RDTs.^53^ In our study, microscopy demonstrated only moderate sensitivity for detecting *P. falciparum* (59.2%) and *P. vivax* (62.0%) monoinfections, with notable difficulty distinguishing mixed infections. This raises concerns around misclassification of *P. vivax*, which may result in a lack of primaquine administration for radical liver cure. The PAN-pLDH/Pf-HRP2 RDT showed strong performance in detecting *P. falciparum* and *P. vivax*, reinforcing its utility in field settings. However, our molecular diagnostics identified a high proportion of submicroscopic (30%) and *Plasmodium* genus of undetermined species (25%) in mainland sites, likely because of the enhanced sensitivity of the reverse transcriptase real-time PCR workflow with a lower limit of detection in the *Plasmodium* genus compared with species-specific assays.^35^ However, we cannot exclude the possibility of false-positive results arising from sample inhibitors, degraded RNA, or other assay-related factors. The qRT-PCR assay applied in this study has been shown to detect both zoonotic and nonzoonotic malaria at up to 10,000-fold-higher threshold limits, providing valuable insights into low-level zoonotic malaria transmission dynamics and supporting malaria elimination efforts in Southeast Asia. Integrating a reverse transcription step into conventional PCR assays has also been demonstrated to significantly improve sensitivity and may represent a more robust approach for complementary molecular surveillance, including in asymptomatic populations at risk.^35^ In contrast, conventional point-of-care tests failed to detect the low-density *P. knowlesi* infections found in patients on Mursala Island, despite their symptomatic presentation. This aligns with previous studies showing poor performance of PAN-pLDH-based RDT for *P. knowlesi*,^13^^,^^14^ although the sensitivity of more recent RDTs has improved for parasite counts greater than 200/*µ*L.^12^ These limitations emphasize the critical need to integrate molecular tools into surveillance systems in areas approaching malaria elimination, especially where *P. knowlesi* is suspected.^30^

*Plasmodium knowlesi* infection has been associated with environmental and occupational risk factors, including forest and agricultural work (particularly among farmers and plantation workers), sleeping outside, and household characteristics such as open eaves or gaps in walls.^9^ In our study, although the majority of *P. knowlesi* cases (71%) were engaged in agricultural activities, this occupation alone did not significantly increase infection risk. Recent forest exposure, house proximity to rubber plantations, and wood collection in forested areas were commonly identified for the small number of *P. knowlesi* infections in Mursala Island, although we were underpowered to determine if these were also statistically significant associations in this setting. It is likely that these trends indicate a zoonotic transmission cycle involving forest-dwelling vectors and macaque reservoirs, contrasting with the coastal semiurban environments of our mainland study sites where *P. knowlesi* infections were not detected. Furthermore, although malaria prevention measures, such as bed net use and personal insect repellent use, may continue to provide benefits for preventing nonzoonotic malaria, their use did not provide significant protection against *P. knowlesi* infection in our study. This finding emphasizes the need for tailored prevention strategies targeting high-risk groups engaged in forest-related activities to effectively mitigate zoonotic malaria transmission risk.

Our study has several limitations. In the mainland study sites, there was an overrepresentation of young individuals, with only 14% of participants being adult men older than 30 years old; additionally, only 3% of all participants reported either agricultural occupations or recent forest exposure. This might have introduced a degree of selection bias by insufficiently capturing high-risk populations, particularly adult forest and agricultural workers who are most vulnerable to zoonotic malaria.^9^ The study's design focus on symptomatic cases presenting to health facilities potentially underestimates the true infection burden by missing mild and asymptomatic cases that did not seek medical attention.^54^ Additionally, the COVID-19 pandemic interrupted both the study timeline and routine malaria activities, potentially affecting health care-seeking behavior and contributing to increased malaria burden during the study period. On Mursala Island, the cross-sectional nature of the surveys and the relatively small number of *P. knowlesi* cases limit our ability to draw definitive conclusions about risk factors and transmission patterns. Furthermore, the lack of laboratory facilities for comprehensive assessments of WHO severity criteria prevented thorough evaluation of key parameters of severe malaria, particularly in *P. knowlesi* cases.

## CONCLUSION

Our study highlights the complex malaria epidemiology in North Sumatra and the ongoing challenges to Indonesia’s elimination goals. Despite control efforts, nonzoonotic malaria—particularly *P. vivax*—remains a major cause of febrile illness on the mainland. In contrast, *P. knowlesi* transmission on Mursala Island reflects distinct zoonotic dynamics linked to forest-related activities, such as rubber plantation proximity, wood collection, and recent forest travel. These findings underscore the need to strengthen molecular diagnostic capacity and adopt improved sampling strategies in future surveillance to better understand and mitigate the growing threat of zoonotic malaria in Indonesia.

## Supplemental Materials

10.4269/ajtmh.25-0493 Supplemental Materials
