# Supplementary material for: Submicroscopic Burden of Zoonotic Plasmodium knowlesi Malaria on Mursala Island and Plasmodium falciparum and Plasmodium vivax Transmission in Mainland North Sumatra, Indonesia
Source: Am J Trop Med Hyg. 2025 Dec 4;114(2):292–301. doi: 10.4269/ajtmh.25-0493 (PMC12874815; doi:10.4269/ajtmh.25-0493)
Supplement: Supplemental Materials [file tpmd250493.SD1.pdf]

Supplemental Table 1 – Epidemiological factors associated with *P. falciparum* and *P. vivax* infections on mainland sites

**Patient characteristic**	**North Sumatra – mainland sites**					
	P. falciparum	***P. vivax***	***P. falciparum /P. vivax***	***Plasmodium* genus**	***Control*** *(negative)*	***P-value*** *(across groups)*
Number enrolled	71	166	5	79	626	
Occupation						
Agriculture	2 (2.8)	5 (3.1)	0 (0)	2 (2.5)	25 (4.0)	0.908
Fisherman	7 (9.9)	8 (4.9)	0 (0)	0 (0)	28 (4.5)	0.071
Household/family carer	7 (9.9)	16 (9.8)	0 (0)	11 (13.9)	93 (14.9)	0.328
No work/unemployed	7 (9.9)	13 (7.9)	0 (0)	10 (12.7)	66 (10.6)	0.713
Other	10 (14.1)	25 (15.2)	3 (60)	7 (8.9)	100 (16.0)	**0.032**
Self-employed/business	2 (2.8)	8 (4.9)	0 (0)	5 (6.3)	50 (8.0)	0.342
Student	36 (36)	89 (54.2)	2 (40)	44 (55.7)	262 (42.0)	**0.016**
Activities						
Sleep outside house (last 2 weeks)	11 (15.5)	33 (20.1)	0 (0)	9 (11.4)	58 (9.3)	0.003
Bednet use outside house	1 (1.4)	5 (3.1)	0 (0)	2 (2.5)	6 (1.0)	0.334
Aware of monkeys	31 (43.7)	59 (36.0)	3 (60)	16 (20.3)	193 (30.9)	**0.012**
Clearing forest/vegetation	8 (11.3)	10 (6.1)	0 (0)	7 (8.9)	32 (5.1)	0.218
Forest exposure (>4 hours)	4 (5.6)	5 (3.1)	1 (20)	4 (5.1)	14 (2.2)	0.058
Cutting timber	0 (0)	0 (0)	0 (0)	0 (0)	2 (0.3)	0.906
Collecting wood	0 (0)	2 (1.2)	0 (0)	0 (0)	4 (0.6)	0.764
Hunting	0 (0)	0 (0)	0 (0)	0 (0)	1 (0.2)	0.972
Charcoal making	0 (0)	0 (0)	0 (0)	1 (1.3)	1 (0.2)	0.313
Other forest activity	1 (1.4)	3 (1.8)	0 (0)	0 (0)	3 (0.5)	0.378
Malaria prevention						
Bed net use (any)	51 (71.8)	120 (73.2)	5 (100)	59 (74.7)	419 (64.1)	0.197
Bed net use (Insecticide treated)	19 (26.8)	35 (21.3)	2 (40)	16 (20.3)	179 (28.3)	0.210
Personal insect repellent (lotion/spray)	9 (12.7)	22 (13.4)	0 (0)	6 (7.6)	114 (18.3)	0.064
Household						
Wall construction						
Wood	18 (25.3)	50 (30.5)	1 (20)	45 (57.0)	185 (29.7)	**<0.001**
Concrete	13 (18.3)	34 (20.7)	1 (20)	14 (17.7)	253 (40.5)	**<0.001**
Tin	1 (1.4)	3 (1.8)	0 (0)	2 (2.5)	1 (0.2)	0.052
Bricks	30 (42.3)	67 (40.9)	3 (60)	17 (21.5)	164 (26.3)	**<0.001**
Bamboo	5 (7.0)	0 (0)	0 (0)	0 (0)	3 (0.5)	**<0.001**
Other	4 (5.6)	10 (6.1)	0 (0)	1 (1.3)	18 (2.9)	0.179
Elevated (>1m stilts)	19 (26.8)	18 (11.0)	1 (20)	19 (24.1)	121 (19.4)	**0.026**
Open eaves/gaps	35 (49.3)	85 (51.8)	2 (40)	34 (43.0)	203 (32.5)	**<0.001**
Insecticide treated walls (IRS)	4 (5.6)	8 (4.9)	0 (0)	3 (3.8)	32 (5.1)	0.964
Household environment (±100m)						
Oil palm	28 (39.4)	57 (34.8)	3 (60)	19 (24.1)	215 (34.5)	0.199
Rubber	1 (1.4)	7 (4.3)	0 (0)	0 (0)	18 (2.9)	0.367
Rice paddy	3 (4.2)	18 (11.0)	3 (60)	4 (5.1)	55 (8.8)	**<0.001**
Mangrove	14 (19.7)	25 (15.2)	2 (40)	6 (7.6)	48 (7.7)	**<0.001**
Cleared forest area	9 (12.7)	9 (5.5)	2 (40)	3 (3.8)	20 (3.2)	**<0.001**

Intact forest area	13 (18.3)	16 (9.8)	2 (40)	5 (6.3)	13 (12.8)	**0.046**

Supplemental Table 2 – Epidemiological factors associated with *P. knowlesi* infections on Mursala Island

**Patient characteristics**	***P. knowlesi***	***Control (malaria negative)***	***Odds ratio (95% CI)***	***P-value***
Number enrolled	7	55		
Occupation				
Agriculture	5	22	4.35 (0.47-40.4)	0.196
Fisherman	1	10	1.82 (0.01-31.9)	0.683
Unemployed	1	19	Reference	-
Activities				
Sleep outside house (last 2 weeks)	1 (14.3)	14 (25.5)	0.49 (0.05-4.41)	0.523
Bednet use outside house	1 (14.3)	0 (0)	3.42 (0.38-30.5)	0.270
Aware of monkeys	6 (85.7)	39 (70.9)	2.46 (0.27-22.1)	0.421
Clearing forest/vegetation	0 (0)	11 (20)	-	0.334
Forest exposure (>4 hours)	6 (85.7)	25 (45.5)	7.13 (0.81-63.9)	0.076
Malaria prevention				
Bed net use (any)	6 (85.7)	35 (63.6)	3.43 (0.38-30.5)	0.270
Personal insect repellent use (lotion/spray)	0 (0)	2 (3.6)	-	0.608
Household				
Wooden walls	7 (100)	51 (92.7)	-	0.999
Elevated (>1m stilts)	4 (57.1)	20 (36.4)	2.33 (0.47-11.49)	0.298
Open eaves/gaps	4 (57.1)	40 (72.7)	0.50 (0.10-2.50)	0.399
Insecticide treated walls (IRS)	0 (0)	4 (7.3)	-	0.461-
Household environment (±100m)				
Oil palm	0 (0)	0 (0)	-	-
Rubber	6 (85.7)	25 (42.5)	7.20 (0.81-63.9)	0.076
Rice paddy	0 (0)	1 (1.8)	-	0.719
Mangrove	2 (28.6)	13 (23.6)	1.29 (0.22-7.46)	0.774
Cleared forest area	1 (14.3)	12 (21.8)	0.60 (0.07-5.45)	0.648
Intact forest area	7 (100)	36 (65.5)	-	0.062
